# Inhibition of miR-214-3p Aids in Preventing Epithelial Ovarian Cancer Malignancy by Increasing the Expression of LHX6

**DOI:** 10.3390/cancers11121917

**Published:** 2019-12-02

**Authors:** Changwon Yang, Hee Seung Kim, Soo Jin Park, Eun Ji Lee, Se Ik Kim, Gwonhwa Song, Whasun Lim

**Affiliations:** 1 Institute of Animal Molecular Biotechnology and Department of Biotechnology, College of Life Sciences and Biotechnology, Korea University, Seoul 02841, Korea; ycw117@korea.ac.kr; 2 Department of Obstetrics and Gynecology, Seoul National University College of Medicine, Seoul 03080, Korea; bboddi0311@gmail.com (H.S.K.); soojin.mdpark@gmail.com (S.J.P.); bliss880103@gmail.com (E.J.L.); seikky@naver.com (S.I.K.); 3 Department of Food and Nutrition, Kookmin University, Seoul 02707, Korea

**Keywords:** EOC, miR-214-3p, LHX6, exosome, apoptosis

## Abstract

In human epithelial ovarian cancer (EOC), various miRNAs can function as either oncogenes or tumor suppressor genes. We investigated miRNAs known to be involved in EOC progression and analyzed their expression in tissues and serum-derived exosomes from benign serous cystadenoma, borderline serous tumor, low-grade serous ovarian cancer, and high-grade serous ovarian cancer patients (HGSO). The HGSO group was divided based on the platinum-free interval, which is defined as the duration from the completion of platinum-based chemotherapy to recurrence. We also analyzed the mRNA levels of target genes that candidate miRNAs might regulate in patient tissues. miR-214-3p was highly expressed in tissues and exosomes derived from EOC with high malignancy and also found to regulate the expression of LIM homeobox domain 6 (LHX6) mRNA. Serum exosomal levels of miR-214-3p were significantly increased in platinum-resistant HGSO (25.2-fold, *p* < 0.001) compared to the exosomal expression of benign tumor patients. On transfection of miR-214-3p inhibitor in EOC cells, cell proliferation was inhibited while apoptotic cell death was increased. Collectively, we suggest that miR-214-3p in serum exosomes can be a potential biomarker for the diagnosis and prognosis of ovarian tumor, and its inhibition can be a supportive treatment for EOC.

## 1. Introduction

Most ovarian cancers originate from the ovarian and fallopian tube epithelium. Epithelial ovarian cancer (EOC) is the fourth leading cause of female cancer-related deaths in Western countries [1]. EOC treatment involves cytoreduction surgery followed by platinum-based chemotherapy. Unfortunately, most patients experience cancer recurrence within 12 to 18 months after the first treatment. Therefore, understanding the molecular mechanisms behind the malignant transformation and recurrence of EOC and developing diagnostic markers to predict poor prognosis in EOC patients are important.

MicroRNAs (miRNAs) are a class of small non-coding RNAs that play a role in cell proliferation, apoptosis, and carcinogenesis by regulating target genes [2]. In human cancer, miRNAs can function as either oncogenes or tumor suppressor genes [3,4]. Similarly, in EOC cells, many miRNAs target specific genes and promote cell proliferation or induce apoptosis [5,6]. For example, miR-31 suppresses the *AFF1* gene and inhibits the proliferation and migration of ovarian cancer cells [7]. miR-193a regulates *MCL1* and induces EOC cell apoptosis [8]. In contrast, miR-213-3p can induce drug resistance in ovarian cancer by targeting the *NAV3* gene [9]. Furthermore, miR-21-5p, miR-141-3p, and miR-200a/b/c are also reported to regulate the drug resistance of EOC and correlate with overall survival [10,11,12]. Besides these, miR-214-3p is overexpressed in ovarian cancer and is associated with overall and progression-free survival [13]. 

miRNAs are the most abundant small non-coding RNAs in exosomes; thus, cancer-derived exosomes can be used to predict prognosis based on miRNA expression patterns [14]. miR-214-3p was recently revealed to be overexpressed in myeloma-derived exosomes and to prevent apoptosis [15]. Serum levels of miRNAs, including miR-373, miR-200a, miR-200b, and miR-200c, are known to have the potential to distinguish between benign tumors and EOC [16]. However, little is known about the expression of miRNAs in serum exosomes and their target genes in EOC tissues. Therefore, we decided to screen for miRNAs overexpressed in tissues and serum exosomes derived from EOC patients. We hypothesized that the selected miR-214-3p would promote malignancy by preventing apoptosis and enhancing proliferation. We also examined changes in the proliferative capacity of EOC cells by regulating LIM homeobox 6 (*LHX6*) expression, the predicted target of miR-214-3p.

## 2. Results

### 2.1. miR-214-3p Is Positively Correlated with the Malignancy of Ovarian Tumors in Human Tissues But Not with Drug Resistance

**Figure 1:**
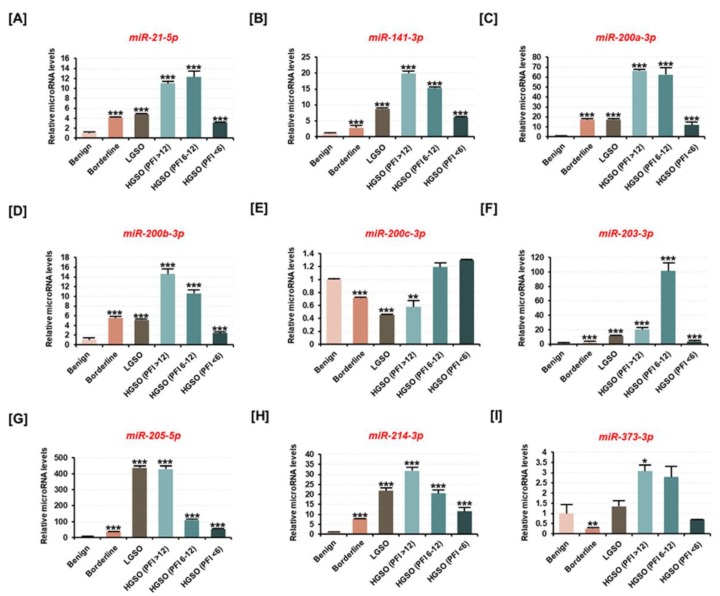
Differences in expression of candidate miRNAs in ovarian cancer patient tissues based on ovarian tumor malignancy. Candidate miRNAs are reported to be associated with epithelial ovarian cancer (EOC) progression, according to previous studies. (**A–I**) The expression of (**A**) miR-21-5p, (**B**) miR-141-3p, (**C**) miR-200a-3p, (**D**) miR-200b-3p, (**E**) miR-200c-3p, (**F**) miR-203-3p, (**G**) miR-205-5p, (**H**) miR-214-3p, and (**I**) miR-373-3p was estimated using miRNA cDNA synthesis and a qPCR kit from the total RNA extracted from tissues of patients with benign tumor, borderline tumor, low-grade serous ovarian cancer (LGSO), high-grade serous ovarian cancer (HGSO) (platinum-free interval (PFI) of >12 months), HGSO (6 months ≤ PFI < 12 months), and HGSO (PFI < 6 months). The HGSO group was subclassified according to the recurrence period after platinum-based chemotherapy. All miRNAs are arranged in numerical order. All experiments were performed in triplicate. The asterisks indicate the significance compared to the benign group (*** *p* < 0.001, ** *p* < 0.01, and * *p* < 0.05).

The total RNA extracted from each tissue was used to measure the expression of nine candidate miRNAs known to be involved in ovarian cancer progression (Figure 1). miRNA analysis revealed that the expression of miR-21-5p, miR-141-3p, miR-200a-3p, miR-200b-3p, miR-203-3p, miR-205-5p, and miR-214-3p was increased in borderline serous tumor, low-grade serous ovarian cancer (LGSO), and platinum-resistant high-grade serous ovarian cancer (HGSO) tissues compared to their expression in benign tumor (Figure 1A–D,F–H). Notably, miR-214-3p expression was increased about 7.9-fold (*p* < 0.001) in borderline tissue, 21.8-fold (*p* < 0.001) in LGSO tissue, and 31.8-fold (*p* < 0.001) in platinum-sensitive HGSO tissue compared to the miR-214-3p expression in benign tissue. However, in partial platinum-sensitive HGSO and platinum-resistant HGSO groups collected after platinum-based chemotherapy, the prognostic effect of miR-214-3p was not verified. However, miR-200c-3p expression was significantly decreased in borderline tissues, LGSO, and platinum-sensitive HGSO (Figure 1E). The differential expression of miR-373-3p with respect to ovarian tumor progression was difficult to determine (Figure 1I). Thus, the results suggested that the expression of miRNAs is significantly altered with respect to ovarian tumor progression and that they can be promoted as potential biomarkers for the diagnosis of EOC.

### 2.2. Target Genes Presumed to Be Regulated by Candidate miRNAs Were Altered by the Malignancy of Ovarian Tissue

**Figure 2:**
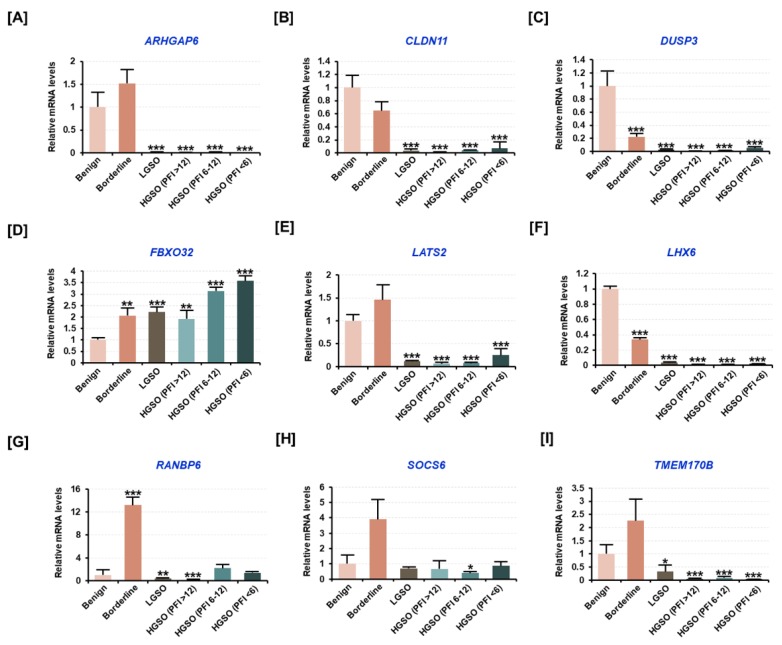
Analysis of the mRNA levels of target genes regulated by the candidate miRNAs in patient tissue samples based on ovarian tumor malignancy. (**A–I**) All genes have been reported to have tumor suppressor functions, according to previous studies. The expression of (**A**) Rho GTPase activating protein 6 (ARHGAP6), (**B**) claudin 11 (CLDN11), (**C**) dual-specificity phosphatase 3 (DUSP3), (**D**) F-box protein 32 (FBXO32), (**E**) large tumor suppressor kinase 2 (LATS2), (**F**) LIM homeobox 6 (LHX6), (**G**) RAN-binding protein 6 (RANBP6), (**H**) suppressor of cytokine signaling 6 (SOCS6), and (**I**) transmembrane protein 170B (TMEM170B) was measured by qPCR analysis from total RNA extracted from tissues of patients with benign tumor, borderline tumor, LGSO, platinum-sensitive HGSO, partial platinum-sensitive HGSO, and platinum-resistant HGSO. The HGSO group was subclassified according to the recurrence period after platinum-based chemotherapy. All genes are arranged in alphabetical order. All experiments were performed in triplicate. The asterisks indicate the significance compared to the benign group (***p < 0.001, ** *p* < 0.01, and * *p* < 0.05).

Further, we measured the mRNA levels of the potential target genes of candidate miRNAs by selecting target mRNAs involved in tumor progression in ovarian tissues using the target prediction database, miRDB (http://mirdb.org). In the earlier studies, the genes which were reported to have tumor suppressor functions were also selected. The analysis revealed that the expression of target genes such as Rho GTPase activating protein 6 (*ARHGAP6*), claudin 11 (*CLDN11*), dual-specificity phosphatase 3 (*DUSP3*), large tumor suppressor kinase 2 (*LATS2*), *LHX6*, Ras-related nuclear protein (RAN)-binding protein 6 (*RANBP6*), suppressor of cytokine signaling 6 (*SOCS6*), and transmembrane protein 170B (*TMEM170B*) tended to decrease in malignant tissues compared to their expression levels in benign tissues (Figure 2A–C,E–I). However, the expression of F-box protein 32 (*FBXO32*) was found to increase with ovarian tumor progression, and its expression was also observed to be increased with a shorter recurrence period after chemotherapy (Figure 2D). Thus, the results suggested that the candidate miRNAs and their respective target genes usually have opposite expression patterns.

### 2.3. The Expression of Ovarian Cancer Patient-Derived Exosomal miR-214-3p Increases with Malignancy

**Figure 3:**
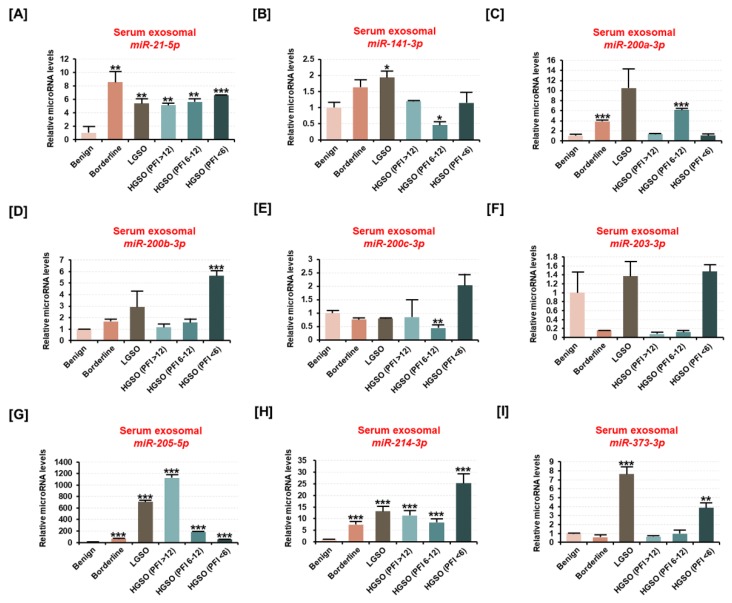
Differences in the expression of candidate miRNAs from serum exosomes derived from ovarian cancer patients based on ovarian tumor malignancy. (**A–I**) The expression of (**A**) miR-21-5p, (**B**) miR-141-3p, (**C**) miR-200a-3p, (**D**) miR-200b-3p, (**E**) miR-200c-3p, (**F**) miR-203-3p, (**G**) miR-205-5p, (**H**) miR-214-3p, and (**I**) miR-373-3p was estimated using miRNA cDNA synthesis and a qPCR kit from the total RNA extracted from serum exosomes of ovarian tumor patients with benign tumor, borderline tumor, LGSO, platinum-sensitive HGSO, partial platinum-sensitive HGSO, and platinum-resistant HGSO. The HGSO group was subclassified according to the recurrence period after platinum-based chemotherapy. All miRNAs are arranged in numerical order. All experiments were performed in triplicate. The asterisks indicate the significance compared to the benign group (*** *p* < 0.001, ** *p* < 0.01, and * *p* < 0.05).

Further, we examined the expression of nine candidate miRNAs after extracting exosomes from the serum of ovarian tumor patients (Figure 3A–I). Exosomal surface proteins such as CD63 and HSP70 were used as evidence for exosome isolation (Figure S1). It revealed that the expression of miR-21-5p, miR-205-5p, and miR-214-3p was significantly increased in exosomes derived from the serum of borderline and serous carcinoma patients compared to the miR expression from benign-tumor-derived exosomes. The expression of the remaining candidate miRNAs (miR-141-3p, miR-200a-3p, miR-200b-3p, and miR-203-3p) was found to be negligible in serous-carcinoma-derived exosomes. Moreover, miR-214-3p expression was significantly increased in exosomes derived from borderline (7.1-fold, *p* < 0.001), LGSO (12.9-fold, *p* < 0.001), platinum-sensitive HGSO (11.2-fold, *p* < 0.001), partial platinum-sensitive HGSO (8.1-fold, *p* < 0.001), and platinum-resistant HGSO (25.2-fold, *p* < 0.001) compared to the exosomal miR-214-3p expression derived from benign tumor patients. Based on the results, we hypothesized that miR-214-3p and its target gene, *LHX6*, are potential candidates in the exosomes that correlate with EOC progression.

### 2.4. Inhibition of LXH6 Increases Proliferating Cell Nuclear Antigen (PCNA) Expression While Inhibition of miR-214-3p Induces Apoptosis on Epithelial Ovarian Cancer Cells

**Figure 4:**
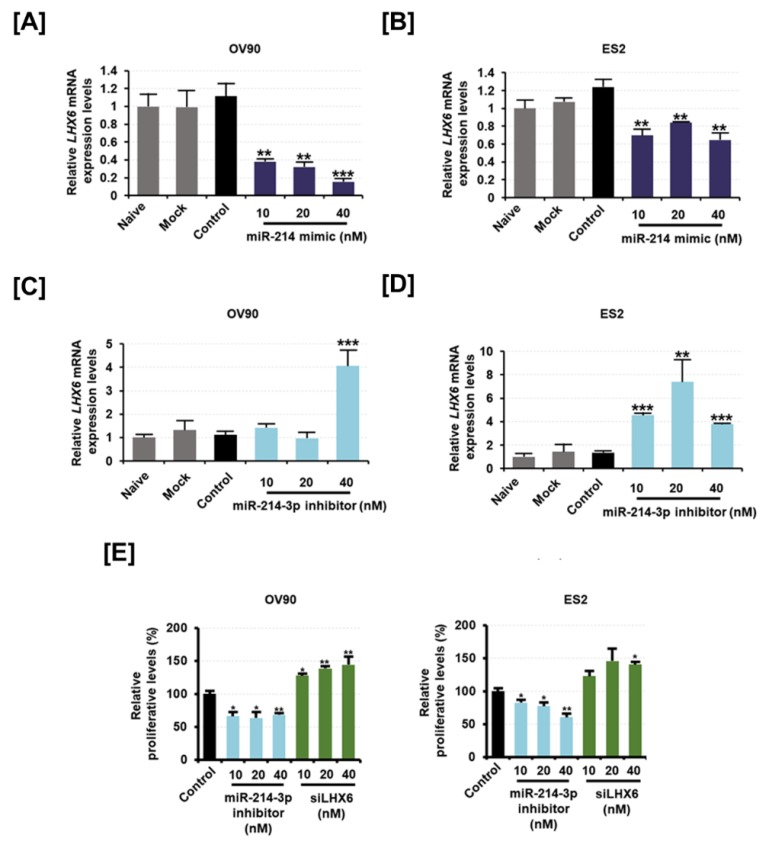
Effect of miR-214-3p inhibitor and LHX6 knockdown on the cell proliferation of OV90 and ES2 cells. (**A,B**) Expression of LHX6 mRNA in (**A**) OV90 cells and (**B**) ES2 cells following transfection of miR-214 mimic (10 nM, 20 nM, and 40 nM). (**C,D**) Expression of LHX6 mRNA in (**C**) OV90 cells and (**D**) ES2 cells following transfection of miR-214-3p inhibitor (10 nM, 20 nM, and 40 nM). (**E**) The dose-dependent effects of miR-214-3p inhibitor (10 nM, 20 nM, and 40 nM) and siLHX6 (10 nM, 20 nM, and 40 nM) on the proliferation of OV90 and ES2 cells were determined, and data are presented as percentages compared to the control (100%). All experiments were performed in triplicate. The asterisks indicate the significance compared to the control (*** *p* < 0.001, ** *p* < 0.01, and * *p* < 0.05).

We artificially regulated miR-214-3p expression in OV90 and ES2 cells using a mimic for miR-214 and an inhibitor for miR-214-3p. We first verified that the miR-214 mimic could inhibit the expression of phosphatase and tensin homolog (PTEN) in EOC, as suggested in a previous study (Figure S2) [17]. The expression of *LHX6* was significantly decreased in miR-214 mimic-transfected OV90 and ES2 cells compared to the *LHX6* expression in control cells (Figure 4A,B). Inversely, when miR-214-3p inhibitor was transfected in OV90 and ES2 cells, *LHX6* expression was significantly elevated (Figure 4C,D). Further, we performed a bromodeoxyuridine (BrdU) incorporation assay to determine whether cell proliferation was regulated by miR-214-3p and *LHX6* expression in EOC cells. We found that the cell proliferation was significantly reduced on transfection of miR-214-3p inhibitor in OV90 as well as in ES2 cells (Figure 4E). However, the proliferation was increased on transfection of siLHX6 in OV90 and ES2 cells. We observed that 40 nM of siLHX6 increased the proliferative levels of OV90 (*p* < 0.01) and ES2 (*p* < 0.05) cells by 1.4-fold as compared to the proliferative levels of corresponding cell-specific control cells.

**Figure 5:**
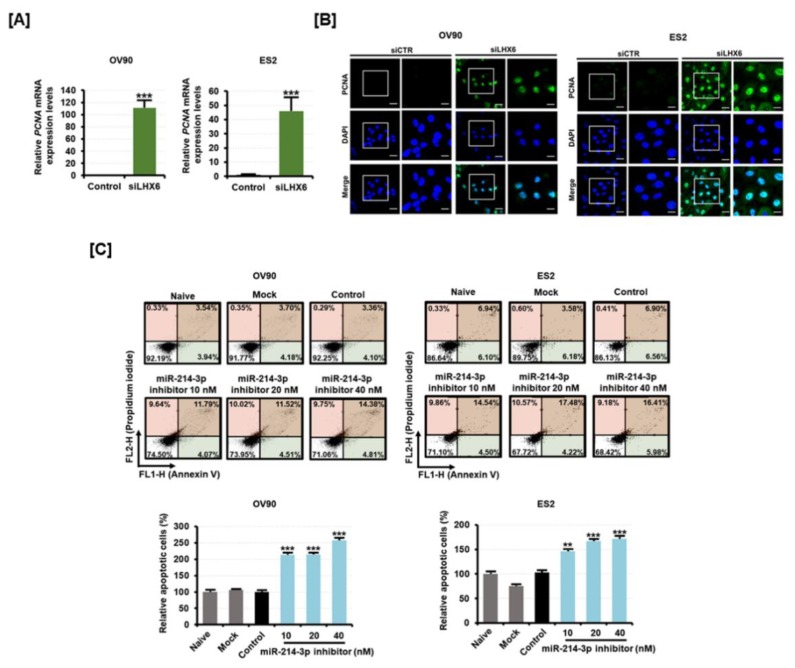
Effect of miR-214-3p inhibitor and LHX6 knockdown on the apoptosis of OV90 and ES2 cells. (**A**) The expression of *proliferating cell nuclear antigen* (*PCNA*) mRNA was measured by qPCR analysis from the RNA of OV90 and ES2 cells transfected with control or siLHX6 (40 nM). (**B**) PCNA protein was detected (green) and the nucleus was counterstained with DAPI (blue) in OV90 and ES2 cells transfected with 40 nM of siLHX6 for 48 h. (**C**) Flow cytometric detection of apoptosis in OV90 and ES2 cells transfected with miR-214-3p inhibitor (20 nM, 40 nM, and 80 nM). Annexin V and propidium iodide (PI) fluorescence values were estimated by flow cytometry. The percentage of apoptotic cells (upper right and lower right quadrants) was analyzed in comparison to the control. All experiments were performed in triplicate. The asterisks indicate the significance compared to the control (*** *p* < 0.001 and ** *p* < 0.01). The scale bar represents 40 μm (in the first and third vertical panels) or 20 μm (in the second and fourth vertical panels).

PCNA is a DNA clamp protein essential for cell proliferation. We found that the mRNA levels of *PCNA* were elevated in response to siLHX6 transfection (Figure 5A). The expression of the PCNA protein was then analyzed by immunofluorescence (Figure 5B). The expression of PCNA was increased in the nuclei of both the EOC cell lines, correlating to the suppression of *LHX6* by siLHX6 transfection. Based on the results, we were able to predict that miR-214-3p can maintain the proliferation of EOC cells while siLHX6 can decrease the proliferation. Moreover, apoptotic cell death was significantly increased when miR-214-3p inhibitor was transfected in OV90 and ES2 cells (Figure 5C). The apoptotic OV90 and ES2 cells were found to increase approximately 2.6-fold (*p* < 0.001) and 1.7-fold (*p* < 0.001), respectively, in response to 40 nM of miR-214-3p inhibitor. The results indicate that miR-214-3p expression increases with tumor malignancy in ovarian-tumor-derived exosomes, whereas inhibition suppresses cell proliferation and promotes apoptosis in EOC.

### 2.5. Inhibition of miR-214-3p Expression Is Involved with the Cell Cycle and Migration of Epithelial Ovarian Cancer Cells

**Figure 6:**
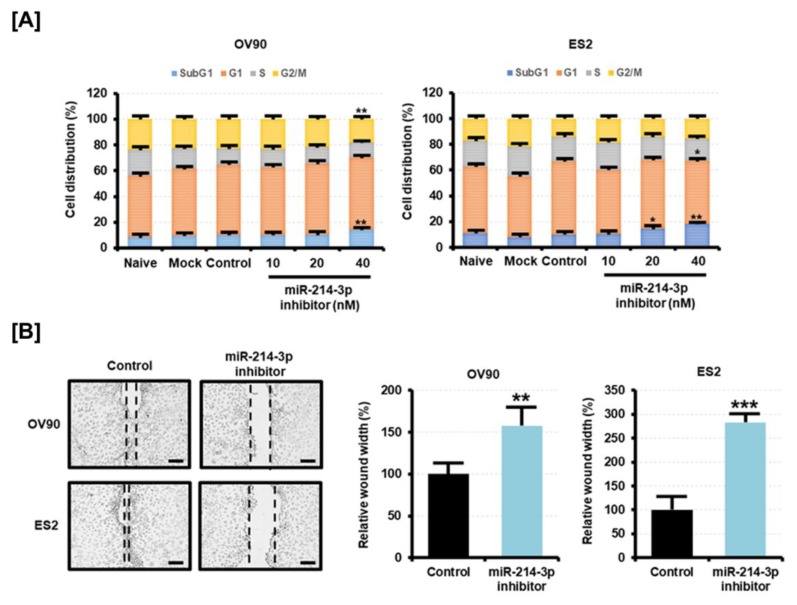
Effect of miR-214-3p inhibitor on the cell cycle and migration capabilities of OV90 and ES2 cells. (**A**) The cell cycle distribution of miR-214-3p inhibitor-transfected OV90 and ES2 cells was detected by flow cytometry. Data are presented as percentages of the cell population in the Sub-G1, G1, S, and G2/M phases. (**B**) The migration of OV90 and ES2 cells on transfection of miR-214-3p inhibitor (40 nM) for 48 h was calculated based on the gap distance and is presented as percentage compared to the control (100%). Images of migrated cells were captured at 10× magnification. The scale bar represents 100 μm. All experiments were performed in triplicate. The asterisks indicate the significance compared to the control (*** *p* < 0.001, ** *p* < 0.01, and * *p* < 0.05).

Using PI staining, we also examined whether the inhibition of miR-214-3p expression alters the cell cycle of EOC cells. We found that miR-214-3p inhibitor increased the proportion of OV90 and ES2 cells in the Sub-G1 phase in a dose-dependent manner (Figure 6A). In contrast, the number of OV90 cells in the G2/M phase decreased on transfection of miR-214-3p inhibitor, and the number of ES2 cells in the G1 phase decreased on transfection of the inhibitor. The miR-214-3p inhibitor also reduced the migration of OV90 and ES2 cells (Figure 6B). The distance of gap regions when transfected with the miR-214-3p inhibitor was higher by approximately 1.6-fold (*p* < 0.01) in OV90 cells and by 2.8-fold (*p* < 0.01) in ES2 cells compared to the gap distance in control cells. The results suggested that miR-214-3p can contribute to cell malignancy by regulating the cell cycle and migration.

### 2.6. Mitochondrial Damage Due to Elevated Intracellular ROS Is Induced by the Inhibition of miR-214-3p Expression

**Figure 7:**
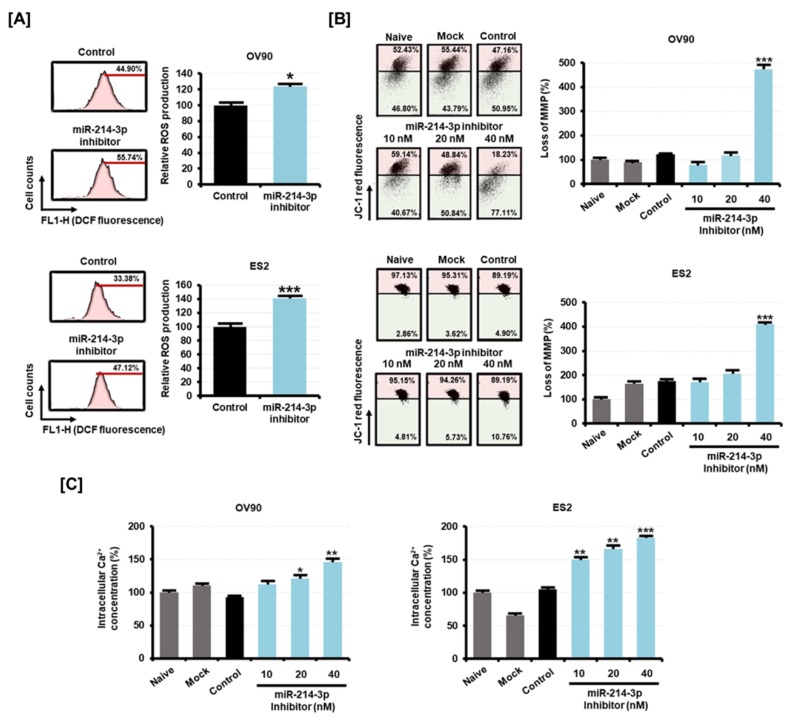
Effect of miR-214-3p inhibitor on the oxidative stress of OV90 and ES2 cells. (**A**) The intracellular reactive oxygen species (ROS) production in response to miR-214-3p inhibitor (40 nM) for 24 h was estimated by flow cytometry. The numbers of 2ʹ,7ʹ-dichlorofluorescein (DCF) green fluorescence-labeled cells represent the relative amounts of intracellular hydrogen peroxides in OV90 and ES2 cells. (**B**) Alteration of the mitochondrial membrane potential (MMP) on transfection of miR-214-3p inhibitor (10 nM, 20 nM, and 40 nM) in OV90 and ES2 cells for 48 h was detected using flow cytometry, and JC-1 staining levels were quantified as the relative ratio of the lower right quadrant to the upper right quadrant. (**C**) Flow cytometric detection of intracellular Ca^2+^ concentration in OV90 and ES2 cells on transfection of miR-214-3p inhibitor (10 nM, 20 nM, and 40 nM). Data are presented as percentages compared to the control (100%). All experiments were performed in triplicate. The asterisks indicate the significance compared to the control (*** *p* < 0.001, ** *p* < 0.01, and * *p* < 0.05).

A tremendous increase in oxidative stress is a typical mechanism leading to the death of cancer cells. After transfection of miR-214-3p inhibitor in OV90 and ES2 cells, we measured the changes in the ROS production by the 2’,7’-dichlorofluorescein diacetate (DCFH-DA) treatment method. The results showed that miR-214-3p inhibitor significantly increased the ROS production in OV90 (1.2-fold, *p* < 0.05) and ES2 cells (1.4-fold, *p* < 0.001) as compared to the ROS levels in control cells (Figure 7A). An excessive increase of ROS causes mitochondrial dysfunction, which leads to the loss of mitochondrial membrane potential (MMP). miR-214-3p inhibitor stimulated a loss of MMP in EOC cells compared to the MMP levels in the control cells (Figure 7B). We observed that transfection of 40 nM of miR-214-3p inhibitor induced MMP losses of 4.7-fold (*p* < 0.001) and 4.1-fold (*p* < 0.001) in OV90 and ES2 cells, respectively. This mitochondrial disorder results from excessive intracellular Ca^2+^ uptake which is stimulated by oxidative stress. OV90 and ES2 cells were transfected with miR-214-3p inhibitor and their intracellular Ca^2+^ concentration was estimated by fluo-4 staining. We found that the intracellular Ca^2+^ concentration was elevated by 1.5-fold (*p* < 0.01) in OV90 cells and 1.8-fold (*p* < 0.001) in ES2 cells with 40 nM of miR-214-3p inhibitor (Figure 7C). The data suggested that the EOC cell death by miR-214-3p inhibitor is mediated by increased oxidative stress.

### 2.7. Inhibition of LHX6 Induces Resistance to Cisplatin in Epithelial Ovarian Cancer Cells

**Figure 8:**
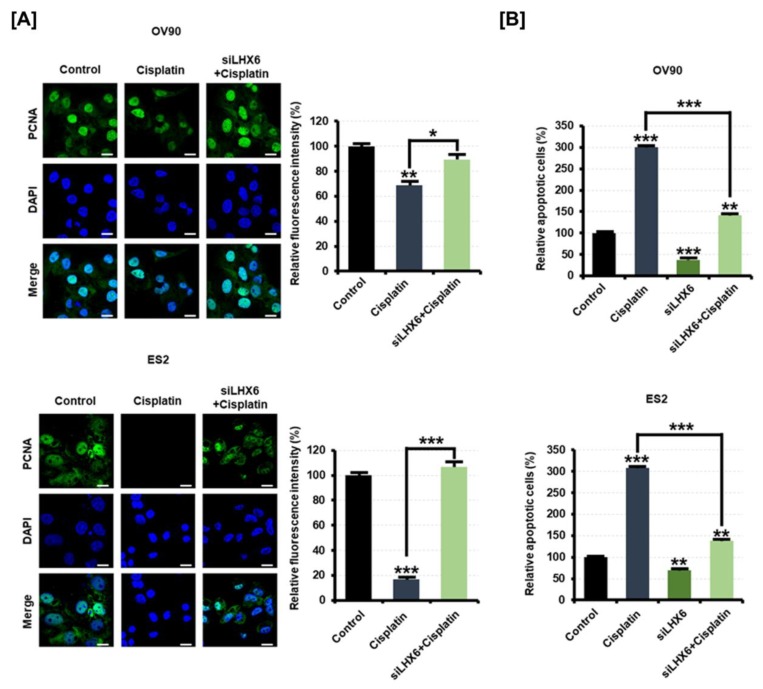
Effect of siLHX6 and cisplatin on the apoptosis of OV90 and ES2 cells. (**A**) PCNA protein was detected (green) and the nucleus was counterstained with DAPI (blue) in OV90 and ES2 cells transfected with 40 nM of control oligonucleotide or siLHX6 for 48 h with cisplatin (10 μM) treatment. The scale bar represents 20 μm. The relative fluorescence intensity was measured as the green/blue ratio using MetaMorph software. (**B**) Flow cytometric detection of apoptosis in OV90 and ES2 cells transfected with siLHX6 (40 nM) and treated with cisplatin (10 μM) for 48 h. Annexin V and PI fluorescence values were estimated by flow cytometry. All experiments were performed in triplicate. The asterisks indicate the significance compared to the control (*** *p* < 0.001, ** *p* < 0.01, and * *p* < 0.05).

Next, we examined the expression of PCNA after treatment with cisplatin with siLHX6 transfected into EOC cells in order to test whether resistance to platinum-based chemotherapy is due to low expression of *LHX6*. As a result, PCNA was decreased by cisplatin in EOC cells transfected with the control, but cells transfected with siLHX6 did not exhibit antiproliferative effects by cisplatin (Figure 8A). When the fluorescence intensity for PCNA was quantified, siLHX6 elevated the intensity on paclitaxel-treated OV90 cells by 1.3-fold (*p* < 0.05) and on ES2 cells by 6.1-fold (*p* < 0.001). In addition, inhibition of *LHX6* also reduced the rate of apoptosis on EOC cells induced by cisplatin (Figure 8B). Under cisplatin treatment, the apoptosis of OV90 cells transfected with siLHX6 was about 52.8% (*p* < 0.001) less than the apoptosis of control oligonucleotide-transfected cells. Moreover, the apoptosis of ES2 cells induced by cisplatin decreased by 55.5% (*p* < 0.001) when *LHX6* was inhibited. These results suggest that the low expression of *LHX6* measured in EOC patient tissues would induce resistance to chemotherapy.

## 3. Discussion

In most women, EOC is diagnosed only at an advanced stage with distant metastasis because of the deep location of the ovaries in the pelvic cavity and the unclear symptoms. Thus, an in-depth study of EOC biology is needed for diagnosis at an early stage using noninvasive techniques. miR-214 is highly conserved among species and is known to be involved in cell differentiation and tissue development [18]. miR-214 has both oncogenic and tumor-suppressing functions in regulating the characteristics of EOC. Some studies have shown that miR-214 targets the *PTEN* gene and induces cell survival and drug resistance in EOC cells [19]. It has also been reported that cell viability and proliferation is increased when miR-214 mimic is transfected in EOC cells [17]. Another study reported that miR-214 overexpression suppressed cell proliferation and induced apoptosis by negative control of the semaphorin-4D gene in EOC cells [20]. Moreover, miR-214 targets β-catenin and can serve as a tumor suppressor for EOC cells [21]. miR-214 has already served as a potential biomarker in different types of cancer [22]. To diagnose EOC using miR-214, resolving the controversy about its bidirectional function is essential. miR-214-3p, derived from the 3′ arm of the pre-miRNA, appears to target a variety of tumor suppressor genes based on the miRNA database. However, the role of miR-214-3p in the regulation of target genes and in their corresponding characteristic changes in EOC cells is still unclear. *LHX6* and *FBXO32*, analyzed in this study, were also expected to be targets of miR-214-3p. In gastric cancer, the inhibition of miR-214 up-regulates *LHX6* and improves resistance to erlotinib [23]. Moreover, high *FBXO32* expression was found to reflect higher five-year overall survival of patients with colorectal cancer [24]. *FBXO32* is also known to be inactivated in gastric cardia adenocarcinoma and esophageal squamous cell carcinoma [25,26]. However, in this study, we found that *FBXO32* expression was elevated with tumor progression in tissues of EOC patients. This suggests that *FBXO32* may have an oncogenic function in EOC, and further studies are needed in this context. In the present study, miR-214-3p expression was high and *LHX6* mRNA expression was low in the tissues of EOC patients with high malignancy.

An exosome is a small-sized membrane vesicle which further enhances miRNA function by transporting miRNAs from the donor cells to the recipient cells. If miR-214-3p is found in serum exosomes and reflects the pathological properties of the cells, it may be possible to diagnose EOC using easier and faster techniques than isolating biopsy tissues. In an earlier study, the expression levels of miR-214, miR-203, miR-205, and miR-73 from ovarian cancer-derived exosomes were reported to be higher compared to their exosomal expression levels in benign tumor [27]. Moreover, it has been shown that RNA-induced silencing complex (RISC)-loading complex (RLC) is also formed within exosomes and has the ability to produce mature miRNAs that are cell-independent. This is possible as proteins involved in miRNA processing, such as Dicer, AGO2, and TRBP, are present within the exosome. The study further shows that exosomes derived from the serum of breast cancer patients convert nontumorigenic epithelial cells into tumors in a Dicer-dependent manner [28]. In our study, we did not evaluate the effect of exosomes on tumor progression. Further investigations are needed to study the role of miR-214-3p overexpression in exosomes promoting the development of EOC. This will help us to verify the therapeutic effect of miR-214-3p in the regulation of EOC. Moreover, exosomes derived from EOC promote the progression of EOC by interacting with immune-related stromal cells such as macrophages in the tumor microenvironment [29]. For example, miR-21-3p, miR-125b-5p, and miR-181d-5p are highly expressed in exosomes derived from EOC, which are involved in M2 polarization of macrophages in hypoxic conditions [30]. Macrophages modified by exosomes promote the proliferation and migration of EOC [31]. The expression and function of exosomal miR-214-3p identified in this study suggest that miR-214-3p may contribute to the oncogenic effects of exosomes by affecting other cell types in the tumor microenvironment of EOC.

*LHX6* mRNA, a member of the LHX6 gene family, encodes a LIM homeodomain transcription factor and can act as a novel cancer biomarker and therapeutic target [32]. *LHX6* inhibits cell proliferation and invasion when overexpressed in breast cancer cells [33,34]. Moreover, hypermethylation of *LHX6* is not found in normal lung tissues; however, it is observed in more than 50% of primary lung cancer. *LHX6* knockdown has been reported to increase cell proliferation, inhibit apoptosis, and cause cell cycle arrest. Here, *LHX6* knockdown increased the proliferation of EOC cells. Immunofluorescence assay showing an increase in PCNA expression on siLHX6 transfection confirmed these findings.

Several studies have reported that the production of ROS in inflammatory sites damages DNA, proteins, and lipids, thereby promoting carcinogenesis [35]. The cause of serous ovarian carcinoma is still unclear. One possibility is stimulation due to genetic defects caused by inflammation because of repeated ovulation in the epithelium of the ovaries and fallopian tubes [36]. Thus, an elevation in ROS production can instigate cancer. However, excessive production of ROS through external stimulation can lead to cancer cell death due to high levels of ROS in cancer cells compared to the ROS levels in normal cells [37]. We previously demonstrated that the induction of ROS production and lipid peroxidation by external stimuli is a mechanism to induce anticancer effects in EOC cells [38,39]. Furthermore, EOC cells are reported to protect themselves through well-organized mitochondrial biomass and are thereby resistant to chemotherapy [40]. Mitochondria are involved in the metabolic remodeling of cancer cells by ROS production and Ca^2+^ ion influx. Moreover, mitochondrial dysfunction due to increased intracellular Ca^2+^ ions is a typical feature of the therapeutic mechanism of cancer cells leading to apoptosis [41]. In this study, we found that the suppression of miR-214-3p promoted ROS production and caused MMP loss with increasing Ca^2+^ concentration in EOC cells. It is clear that the inhibition of miR-214-3p is associated with the molecular mechanisms that inhibit proliferation and migration while inducing migration via regulation of the *LHX6* gene. This study also revealed that the inhibition of *LHX6* in EOC cells is involved in resistance to platinum-based chemotherapy. This suggests that LHX6 may be a novel target for improving drug resistance in anticancer studies, along with a previous study that showed that down-regulation of *LHX6* can induce resistance to chemotherapy in lung cancer [23].

## 4. Materials and Methods

### 4.1. Cell Culture

OV90 and ES2 ovarian cancer cells were purchased from the American Type Culture Collection (Manassas, VA, USA) and maintained in McCoy’s 5A Medium (Cat No: 16600-082, Gibco, Waltham, MA, USA) with 10% fetal bovine serum at 37 °C in a CO_2_ incubator. For the experiments, monolayer cultures of OV90 and ES2 cells were grown in the culture medium to about 70% confluency in 100 mm tissue culture dishes.

### 4.2. Human Tissues and Serum

Ovarian cancer tissues were collected from 29 patients with advanced ovarian cancer after primary debulking surgery and frozen in liquid nitrogen at −80 °C. The collected biopsies were benign serous cystadenoma (*n* = 5), borderline serous tumor (*n* = 4), low-grade serous ovarian cancer (LGSO; *n* = 5), or high-grade serous ovarian cancer (HGSO; *n* = 15). Further, the HGSO biopsies were divided according to their platinum-free interval (PFI), which is defined as the duration from the completion of platinum-based chemotherapy to recurrence. The PFI-based subclassification of HGSO tissues was as follows: platinum-resistant (PFI < 6 months; *n* = 5); partial platinum-sensitive (6 months ≤ PFI < 12 months; *n* = 5); platinum-sensitive (PFI > 12 months; *n* = 5). Blood samples were collected from the same patients before surgery, and the peripheral blood was centrifuged at 4 °C at 1300*g* for 10 min to obtain matched serum and stored at −80 °C. All tissues and blood samples were obtained from Seoul National University Hospital Human Biobank, and the isolated matched serum was stored in the same facility. The current study was approved by the Institutional Review Board of Seoul National University Hospital (No. 1708-053-876). 

### 4.3. RNA Extraction and Quantitative RT-PCR

The total cellular RNA was isolated using Trizol reagent (Invitrogen, Waltham, MA, USA) according to the manufacturer’s instructions. The complementary DNA (cDNA) was synthesized using the total RNA (1 µg) and AccuPower® RT PreMix (Bioneer, Daejeon, Korea). The gene expression levels were measured using SYBR® Green (Sigma, St. Louis, MO, USA) and the StepOnePlus™ Real-Time PCR System (Applied Biosystems, Foster City, CA, USA). The primers used for cDNA synthesis are listed in Table 1. Sequence-specific products were detected by generating a melting curve. The CT value represents the cycle number corresponding to a fluorescent signal that was statistically higher compared to the background signal. The relative gene expression was quantified using the 2^−ΔΔCT^ method.

### 4.4. Quantitative RT-PCR Analysis for microRNA 

For miRNA detection, the first-strand cDNA was synthesized from the total RNA using a miRNA first-strand cDNA synthesis kit (Agilent Technologies, Santa Clara, CA, USA). The target miRNAs were designed as shown in Table 2, and miRNAs expression was measured using the High-Specificity miRNA QPCR Core Reagent Kit (Agilent Technologies, Santa Clara, CA, USA) following the manufacturer’s protocol. For internal control, U6 small nuclear RNA (snRNA) was used to standardize the cDNA template variations within the target samples as previously described in studies with ovarian tissues and serum [6,42,43,44]. The relative quantification of miRNAs was determined using the 2^−ΔΔCT^ method.

### 4.5. Exosome Isolation

Approximately 100 mL of culture medium was centrifuged at 3000*g* for 15 min to remove intact cells and debris while the supernatant was collected. The supernatant medium was centrifuged at 4000*g* using Amicon® Ultra-15 Centrifugal Filter Units (Cat No: C7715, Merck, Kenilworth, NJ, USA) to obtain a concentrate. The exosomes in the concentrated culture medium were extracted from the cell culture medium using a Total Exosome Isolation Kit (Cat No: 4478359, Thermo Fisher Scientific, Waltham, MA, USA) according to the manufacturer’s instructions. Briefly, the culture medium was mixed with 0.5 mL of isolation reagent and incubated overnight at 4 °C. Further, the mixture was centrifuged at 4 °C and at 10,000*g* for 1 h, and the supernatant was collected. To extract the exosomal RNA and proteins from the supernatant, a Total Exosome RNA and Protein Isolation Kit (Cat No: 4478545, Thermo Fisher Scientific) was used according to the manufacturer’s instructions. Antibodies against HSP70 and CD63 were purchased from System Biosciences (Palo Alto, CA, USA) and used as surface protein markers to confirm the exosome isolation by Western blot.

### 4.6. Transfection of microRNA Mimics, Inhibitors, and siRNA

For the transfection of miRNA mimics, inhibitors, and small interfering RNA (siRNA), OV90 and ES2 cells were seeded in 6-well culture plates and transfected with nontargeting control, miRNA mimics, inhibitors, and siRNA using transfection reagent Lipofectamine 2000 according to the manufacturer’s instructions. All RNA oligonucleotides were synthesized by Bioneer (Daejeon, Korea). Briefly, oligonucleotides and Lipofectamine 2000 were diluted in Opti-MEM reduced serum medium (Cat No: 32985070, Gibco, Waltham, MA, USA) and added to the cells. After 6 h of incubation at 37 °C in a CO_2_ incubator, the medium was replaced with serum-containing medium.

### 4.7. Proliferation Assay

Proliferation assays were conducted using a Cell Proliferation ELISA, BrdU kit (Cat No: 11647229001, Roche, Indianapolis, IN, USA) according to the manufacturer’s protocol. Briefly, 10 µM of bromodeoxyuridine (BrdU) was added to the cultured cells and incubated for an additional 2 h at 37 °C. The cells were fixed and incubated with anti-BrdU-peroxidase (POD) working solution for 90 min. The anti-BrdU-POD binds to the BrdU incorporated into newly synthesized cellular DNA and is then detected by 3,3′,5,5′-tetramethylbenzidine (TMB) substrate. The reaction product was quantified by measuring the absorbance at 370 nm and 492 nm using an ELISA reader.

### 4.8. Immunofluorescence Microscopy

The effect of inhibitors against siLHX6 on the expression of proliferating cell nuclear antigen (PCNA) in OV90 and ES2 cells was determined using immunofluorescence microscopy as described previously [45]. Cells were transfected with siLHX6 (40 nM) for 48 h, and the experiment was performed in triplicate. Images were captured using an FV3000 confocal microscope (Olympus, Tokyo, Japan) equipped with a digital microscope camera and FV3000 RS fluoview software.

### 4.9. Determination of Apoptosis by Annexin V and Propidium Iodide (PI) Staining

The induction of apoptosis in OV90 and ES2 cells by miR-214-3p inhibitor was analyzed using a fluorescein isothiocyanate Annexin V apoptosis detection kit I (BD Biosciences, Franklin Lakes, NJ, USA) as described previously [45].

### 4.10. Cell Cycle Analysis

To examine the distribution of Sub-G1, G1, S, and G2/M phases in miR-214-3p inhibitor-transfected cells, we stained OV90 and ES2 cells with propidium iodide (PI; BD Biosciences, Franklin Lakes, NJ, USA) in the presence of 100 μg/mL RNase A (Sigma, St. Louis, MO, USA). The fluorescence was analyzed using a flow cytometer (Merck, Kenilworth, NJ, USA).

### 4.11. Migration Assay

Cell migration was evaluated using Ibidi migration culture-inserts according to the manufacturer’s instruction (Ibidi, Munich, Germany). A 70 μL suspension of OV90 and ES2 cells (2 × 10^5^ cells/mL) was seeded into each well of the culture-inserts and grown overnight until 100% confluence. The cells were then transfected with 40 nM of miR-214-3p. After 12 h of incubation at 37 °C in a CO_2_ incubator, the migration of cells into the defined cell-free gap (500 μm) was observed, and light microscopy images of the gap field were acquired using a DM3000 (Leica, Wetzlar, Germany). For analysis, the gap closure was computed.

### 4.12. Cellular ROS Determination 

Intracellular reactive oxygen species (ROS) production was estimated using 2’,7’-dichlorofluorescein diacetate (DCFH-DA, Sigma, St. Louis, MO, USA), which converts to fluorescent 2’,7’-dichlorofluorescein (DCF) in the presence of peroxide. Cells were transfected with miR-214-3p inhibitor for 24 h. Cells were then detached using trypsin-ethylenediaminetetraacetic acid, collected by centrifugation, and washed with phosphate-buffered saline. Further, cells were treated with 10 μM DCFH-DA for 30 min at 37 °C and washed twice with PBS, and the DCF fluorescence intensity was analyzed using a flow cytometer (Merck, Kenilworth, NJ, USA). The data represent three independent experiments.

### 4.13. JC-1 Mitochondrial Membrane Potential Assay

The JC-1 mitochondrial membrane potential (MMP) was analyzed using a mitochondria staining kit (Cat No: CS0390, Sigma, St. Louis, MO, USA). Cells were collected by centrifugation and resuspended in a staining solution containing 200× JC-1 and 1× staining buffer. The suspension was incubated at 37 °C in a CO_2_ incubator for 20 min. The stained cells were collected by centrifugation and washed once with 1× JC-1 staining buffer. After washing, the cells were centrifugated again and resuspended in 1 mL staining buffer. The fluorescence intensity was analyzed using a Guava® easyCyte™ flow cytometer (Merck, Kenilworth, NJ, USA).

### 4.14. Measurement of the Intracellular Free Ca^2+^ Concentration

Cells were transfected with miR-214-3p inhibitor at different concentrations (10 nM, 20 nM, and 40 nM) for 48 h at 37 °C in a CO_2_ incubator. The supernatant was removed from the culture dishes, and adherent cells were detached with trypsin-EDTA. Cells were then collected by centrifugation. For intracellular Ca^2+^ analysis, cells were resuspended using 3 μM Fluo-4 AM (Cat No: F14201, Invitrogen, Waltham, MA, USA) and incubated at 37 °C in a CO_2_ incubator for 20 min. The stained cells were washed with PBS. The fluorescence intensity was analyzed using a flow cytometer (Merck, Kenilworth, NJ, USA).

### 4.15. Statistical Analysis

The data were subjected to analysis of variance (ANOVA) according to the general linear model (PROC-GLM) of the SAS program (SAS Institute, Cary, NC, USA) to determine statistically significant differences in response to the treatments. *p* < 0.05 was considered to indicate statistically significant difference. Data are presented as the mean ± standard error of the mean (SEM) unless otherwise stated.

## 5. Conclusions

**Figure 9:**
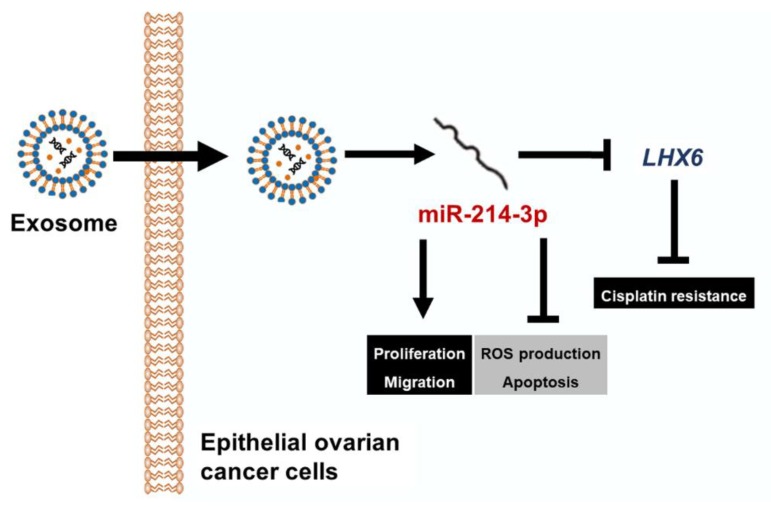
A schematic diagram illustrating the current working hypothesis. miR-214-3p is highly expressed in epithelial ovarian cancer (EOC). The observed oncogenic effects of miR-214-3p include increasing cell proliferation and migration and preventing cell death based on the excessive production of ROS. miR-214-3p which is transferred to the recipient cells by exosomes regulates LHX6 mRNA. When miR-214-3p expression is inhibited, apoptosis is induced by mitochondrial damage due to excessive oxidative stress. Inhibition of LHX6 induces resistance to cisplatin. In summary, miR-214-3p is a predictor of malignancy of ovarian tumors, and its inhibition can synergize with other treatments of EOCs through the modulation of LHX6.

To summarize, we found that among several candidate miRNAs, miR-214-3p detected in the tissues and exosomes is positively correlated with tumor progression in EOC patients. Moreover, expression of *LHX6*, which is presumed to be the mRNA target of miR-214-3p, showed a negative correlation with tumor progression in EOC tissues, as illustrated in Figure 9. The miR-214-3p inhibitor was able to reduce the expression of *LHX6* and also showed anticancer effects in EOC cell lines. The effect of miR-214-3p inhibition seems to be due to the mitochondrial disorders caused by excessive ROS production. Thus, miR-214-3p and *LHX6* may be novel targets in the diagnosis of exosome-based EOC, and altering miR-214-3p expression can play a therapeutic role in EOC.

## Figures and Tables

**Table 1:** Primer sets used in qRT-PCR.

Gene Symbol	Regulatory miRNA	Sense Primer (5′→3′)	Antisense Primer (5′→3′)	Accession Number
*ARHGAP6*	*miR-200b-3p/miR-200c-3p*	TGTCGTCGTCAAAGTCAAGG	CCAGAGGAACCTGTCATTCC	NM_001287242.2
*CLDN11*	*miR-205-5p*	TGGTGTTTTGCTCATTCTGC	GCCTGCATACAGGGAGTAGC	NM_001185056.2
*DUSP3*	*miR-200a-3p*	ATCTCAACGACCTGCTCTCG	GGGTGATGCCTAGTTTCTGC	NM_004090.4
*FBXO32*	*miR-214-3p*	TTGTCCGATGTTACCCAAGG	GCAGCTCTCGGGTTATTGG	NM_001242463.2
*LATS2*	*miR-373-3p*	AGTGACACTTCCCTGGATGC	CCCGATTCATTAGCAAAAGG	NM_014572.3
*LHX6*	*miR-214-3p*	GGACCGATATCTGCTAAGG	CGAATCGGCTGAAGTAGTCC	NM_001242333.2
*RANBP6*	*miR-141-3p*	TTCAGGACTTGAAGCAAAAGC	CCACTCGAACATTGTCATGG	NM_001243202.2
*SOCS6*	*miR-203-3p*	ACCATTGCTACCTCCAATGC	TGACAGCGCATACTTTCAGC	NM_004232.4
*TMEM170B*	*miR-200a-3p*	CGACCACTCCATGATCAACC	GAGAGCCCAGAGGAAGATCC	NM_001100829.3

**Table 2:** miRNA sequences used in miRNA qRT-PCR.

miRNA Name	miRNA Sequence (5′→3′)	NCBI Gene ID of Precursor miRNA
*miR-21-5p*	UAGCUUAUCAGACUGAUGUUGA	406991
*miR-141-3p*	UAACACUGUCUGGUAAAGAUGG	406933
*miR-200a-3p*	UAACACUGUCUGGUAACGAUGU	406983
*miR-200b-3p*	UAAUACUGCCUGGUAAUGAUGA	406984
*miR-200c-3p*	UAAUACUGCCGGGUAAUGAUGGA	406985
*miR-203-3p*	GUGAAAUGUUUAGGACCACUAG	406986
*miR-205-5p*	UCCUUCAUUCCACCGGAGUCUG	406988
*miR-214-3p*	ACAGCAGGCACAGACAGGCAGU	406996
*miR-373-3p*	GAAGUGCUUCGAUUUUGGGGUGU	442918

## Supplementary Materials

The following are available online at https://www.mdpi.com/2072-6694/11/12/1917/s1, Figure S1: Expression of CD63 and HSP70 for the identification of serum exosomes derived from ovarian cancer patients based on ovarian tumor malignancy, Figure S2: Expression of PTEN mRNA in OV90 and ES2 cells following transfection of miR-214 mimic.
