# Supplementary material for: Inhibition of miR-214-3p Aids in Preventing Epithelial Ovarian Cancer Malignancy by Increasing the Expression of LHX6
Source: Cancers (Basel). 2019 Dec 2;11(12):1917. doi: 10.3390/cancers11121917 (PMC6966693; doi:10.3390/cancers11121917)
Supplement: Supplementary file 1 [file cancers-11-01917-s001.pdf]

cancers

Supplementary Materials:

Inhibition of miR-214-3p Aids in Preventing Epithelial Ovarian Cancer Malignancy by Increasing the Expression of LHX6

Changwon Yang †, Hee Seung Kim †, Soo Jin Park, Eun Ji Lee, Se Ik Kim, Gwonhwa Song * and Whasun Lim *

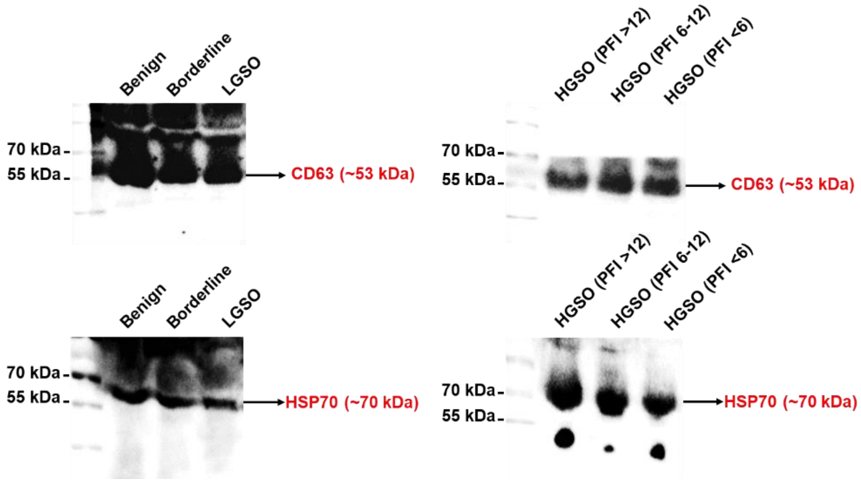

Figure S1. Expression of CD63 and HSP70 for identification of serum exosomes derived from ovarian cancer patients based on ovarian tumor malignancy. Expression of CD63 and HSP70 as exosomal surface proteins was confirmed by western blot analysis. Total Exosome RNA and Protein Isolation Kit was used to extract proteins from exosomes of ovarian tumor patients with benign tumor, borderline tumor, LGSO, platinum sensitive HGSO, partial platinum sensitive HGSO, and platinum resistant HGSO.

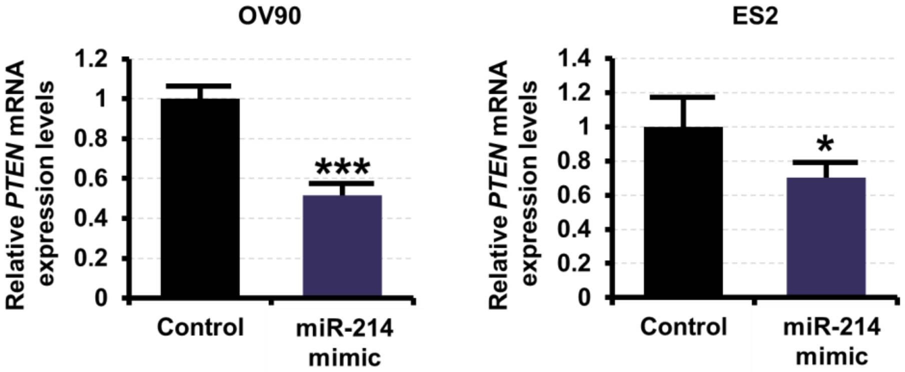

Figure S2. Expression of PTEN mRNA in OV90 and ES2 cells following transfection of miR-214 mimic. Expression of PTEN mRNA was reduced by miR-214 mimic (40 nM) in OV90 and ES2 cells. Primers for PTEN were: (sense 5′-GCC AAC CGA TAC TTT TCT CC-3′) and (antisense 5′-GCT AGC CTC TGG ATT TGA CG-3′). The asterisks indicate the significance compared to control (*** $p < 0.001$ and * $p < 0.05$).
